# The Extracellular Matrix and Neuroblastoma Cell Communication—A Complex Interplay and Its Therapeutic Implications

**DOI:** 10.3390/cells11193172

**Published:** 2022-10-10

**Authors:** Irena Horwacik

**Affiliations:** Laboratory of Molecular Genetics and Virology, Faculty of Biochemistry, Biophysics and Biotechnology, Jagiellonian University, 30-387 Kraków, Poland; irena.horwacik@uj.edu.pl

**Keywords:** extracellular matrix, ECM, neuroblastoma, adhesion, degradation, migration, invasion, metastasis, resistance, cancer therapy

## Abstract

Neuroblastoma (NB) is a pediatric neuroendocrine neoplasm. It arises from the sympatho-adrenal lineage of neural-crest-derived multipotent progenitor cells that fail to differentiate. NB is the most common extracranial tumor in children, and it manifests undisputed heterogeneity. Unsatisfactory outcomes of high-risk (HR) NB patients call for more research to further inter-relate treatment and molecular features of the disease. In this regard, it is well established that in the tumor microenvironment (TME), malignant cells are engaged in complex and dynamic interactions with the extracellular matrix (ECM) and stromal cells. The ECM can be a source of both pro- and anti-tumorigenic factors to regulate tumor cell fate, such as survival, proliferation, and resistance to therapy. Moreover, the ECM composition, organization, and resulting signaling networks are vastly remodeled during tumor progression and metastasis. This review mainly focuses on the molecular mechanisms and effects of interactions of selected ECM components with their receptors on neuroblastoma cells. Additionally, it describes roles of enzymes modifying and degrading ECM in NB. Finally, the article gives examples on how the knowledge is exploited for prognosis and to yield new treatment options for NB patients.

## 1. Introduction

Neuroblastoma is a childhood solid tumor that arises from neural-crest-derived multipotent progenitor cells. Neural crest cells (NCCs) are a population of cells of the neuroepithelial origin that delaminate and extensively migrate from the dorsal neural tube of an embryo to yield, among others, structures of the peripheral nervous system, but also the medulla of the adrenal gland [1]. In light of this, although the quest to identify NB cells of origin is still ongoing, NB can be viewed as an embryonal tumor of the sympatho-adrenal lineage cells that fail to differentiate (see [2] for a review on the topic). The most common primary site of neuroblastoma is the adrenal gland, which is responsible for adrenaline and noradrenaline production by chromaffin cells of its medulla; hence, neuroblastoma is also a neuroendocrine tumor, but it can develop in other locations along the sympathetic axis, such as the mediastinum, pelvis, or neck [3].

Considering the prevalence of children neoplasms, NB is the third after leukemias and central nervous system tumors, and it is the most common extracranial tumor. Moreover, incidence rates of NB are related to the age of children. Hence, it is the most common tumor in infants, with the median age at diagnosis being 18–22 months, and the majority of cases diagnosed in children by the age of 5 [4].

Heterogeneity at presentation is a hallmark of NB. Historically, in view of a wide range of clinical manifestations, several classification systems of NB have been used, applying prognostic factors to assign patients to risk groups to stratify treatment [4,5]. The International Neuroblastoma Pathology Classification (INPC) categorizes peripheral neuroblastic tumors, based on histopathologic factors: the degree of Schwannian development of stroma, the morphology and degree of maturation of neuroblastic cells, and the mitosis–karyorrhexis index (MKI), and defines favorable and unfavorable subgroups [6]. In the past, the International Neuroblastoma Staging System (INSS) has been used, which relies on clinical and radiological assessments of a patient disease and the ability to surgically remove the tumor [7]. Nowadays, a pre-operative staging system, based on pre-treatment “image defined risk factors” (IDRFs), is used to establish, if the tumor can be removed and to predict possible complications during surgery. It is called the International Neuroblastoma Risk Group Staging System (INRGSS) [8]. The INRG classifier includes age of a patient at diagnosis (children of ≥18 months of age have poor prognoses), INGRSS stage, histologic category, grade of differentiation, *MYCN* status (amplification of the *MYCN* oncogene is diagnosed in about 20% of NB patients), ploidy, and the presence or absence of gain in the long arm of chromosome 11. This results in 16 categories of NB with very low risk (>85% 5-year event-free survival, EFS), low risk (from >75% to ≤85% 5-year EFS), intermediate (from ≥50% to ≤75% 5-year EFS), or high risk (<50% 5-year EFS) (see [9] for detail). Ongoing efforts are being made to update the classification criteria [5,10].

The treatment of neuroblastoma is based on risk stratification. Importantly, it can be reduced for low-risk disease. In contrast to an often-aggressive course of HR NB, e.g., children with NB without *MYCN* amplification and 11q aberration grouped in “metastatic special” (i.e., the MS stage of INGRSS—with NB disseminated to skin, liver, and/or restricted bone marrow involvement and age <18 months) have very good prognoses [9]. In some cases, the treatment can be minimal, because NB in the MS group may regress at a high rate despite its disseminated state [11]. However, patients with HR NB are treated with complex, intensive multi-step protocols, consisting of multidrug chemotherapy, surgery, radiotherapy, and the transplantation of autologous stem cells [12]. Furthermore, the treatment of minimal residual disease with a differentiation agent 13-*cis* retinoic acid (RA) and monoclonal antibodies (mAbs) binding GD2 ganglioside (GD2), e.g., dinutuximab [13], dinutuximab beta [14], is applied. However, only about 50% of children will survive event-free 5 years in groups of patients with HR NB [9,12]. Moreover, patients with refractory or recurrent NB are in need of new treatment options [15,16].

Phenomes of NB cells and their underlying genetic and epigenetic background remain to be fully uncovered. Genomic aberrations detected in NB can inform prognoses and include whole-chromosome gains, segmental chromosomal alterations such as losses of 1p, 3p, 4p, and 11q, and gains of 1q, 2p, 7q, 11q13.3, 12q, and 17q [17]. Amplification of *MYCN* is a negative prognostic factor detected in approximately 20% of primary NB tumors and about 50% of HR NB [9,18]. The *MYCN* gene encodes MYCN proto-oncogene (bHLH transcription factor, located on 2p24.3) that regulates cell growth and proliferation, survival, stemness, and differentiation (for a review on the topic, see [19]). The experimental over-expression of *MYCN* in primary NCCs led to their transformation to NB [20]. Consequently, targeting MYCN in NB is a pivotal yet still unaccomplished goal [21]. Other frequently mutated genes in NB include telomerase reverse transcriptase (*TERT*), SH3 and multiple ankyrin repeat domains 2 (*SHANK2*), protein tyrosine phosphatase receptor type D (*PTPRD*), ALK receptor tyrosine kinase (*ALK*), and ATRX chromatin remodeler (*ATRX*) [17]. Additionally, mutations of *ALK* and *PHOX2B* (paired like homeobox 2B) are known to predispose to familial NB [22]. Contemporary research efforts are being applied to characterize phenotypic effects associated with NB genetic mutations and translation of the knowledge to improve the treatment of patients with NB (for a review, see [22]).

Several NB models have been applied to pre-clinical NB research (see the selected reviews below for more detail). These comprise a collection of NB cell lines, characterized with respect to their genetic and phenotypic features. Thus, neuroblastic (N), substrate adherent (S), and intermediate (I) cell types have been described in NB cell lines [23]. Additionally, components of transcriptomic programs and their epigenetic regulation were recently characterized, in attempts to shed more light on the observed heterogeneity and plasticity of NB [24,25]. The cells can be grown in 2D and 3D cultures [26,27]. Patient-derived xenograft (PDX) models are also available [28]. Additionally, animal models that encompass chick embryo [29], zebrafish [30], and various mouse immunodeficient and immunocompetent models have been introduced [31]. The latter include the TH-MYCN transgenic mouse model, in which NB tumors are spontaneously arising and its derivatives, as well as models involving, e.g., subcutaneous (s.c.), orthotopic, into the spleen or intra venous (i.v.) injections of cells [31]. Their application enables the modeling of genetic features of NB, its heterogeneity, analysis of NB cell origins and development, characterization of interactions of NB cells with components of TME, signaling pathways that determine fate of NB cells, and finally, allow for the testing of novel drug candidates.

## 2. Complex Interplay between Tumor Cells and ECM Components in NB

Tumor cells evolve during cancer development and progression in the context of bidirectional crosstalk with the components of TME [32]. In this regard, malignant cells are “immersed” in dynamic interactions with the ECM (the non-cellular TME component), but also fibroblasts, cells forming blood or lymphatic vessels, and immune cells. Functions of stromal cells such as cancer-associated fibroblasts (CAFs) and tumor-associated macrophages (TAMs) are studied in NB tumors, e.g., in relation to NB risk factors [27,33]. Their roles include, e.g., the deposition of ECM structural components, production of degrading and modifying enzymes to affect architecture and stiffness of the ECM, and the release of growth factors (GFs), cytokines, chemokines to regulate not only tumor cell phenotype, but also angiogenesis, inflammation, and immunosuppression (for a detailed characteristic of cellular components of the stroma, including a landscape of immune cells and their properties in the context of NB; readers are directed to recent review articles on the topics [27,33]). This review focuses mainly on the molecular mechanisms and effects of interactions of selected ECM components with their receptors on NB cells.

The “core matrisome” itself gathers about 300 proteins [34]. The ECM components are collagens, proteoglycans (PGs), glycoproteins, ECM-bound growth and other secreted factors, and ECM modifying enzymes [35]. ECM composition is a manifestation of the tissue phenotype, but it is not static, because it can be subjected to remodeling or degradation, in both physiological and pathological processes [36].

### 2.1. ECM Constituents Produced by NB Cells

Earlier studies of Gladson et al. reported that the expression of vitronecin, laminin, collagen positively correlated with the differentiation of peripheral neuroblastic tumors [37]. Thus, vitronectin was detected in normal matured peripheral neurons (i.e., pheochromocytes), as well in ganglion and Schwann cells (SCs) of adult benign ganglioneuroma (GN) tumors. Vitronectin, laminin, fibronectin were mainly absent in undifferentiated NB tumors, and the neuroblast cell population of ganglioneuroblastoma (GNB) tumors, but the proteins were present in gangliocytic cells and their adjacent extracellular matrix, as well as in SCs [37]. Collagen-positive tumor cells (detected with application of trichome and reticulin stains) were also reported—mainly for GNB samples—although no collagen type IV from tumor cells was detected by immunohistochemistry (IHC). Collagen type IV was detected in the SCs of GNB tumors. Finally, collagen type IV, fibronectin, and laminin staining signals were localized to basement membranes of blood vessels of neuroblastic tumors [37].

The findings regarding the aforementioned lack of vitronectin expression in NB tumors were recently opposed, based on digital analyses of large cohorts of patient samples by Burgos-Panadero et al., pointing to a tumor cell origin of vitronectin in NBs and analyzing patterns of vitronectin deposition in relation to NB prognostic factors (see [38] and Section 2.2).

Cell cultures of neuroblastoma cell lines, e.g., CHP-126, IMR-32, and SMS-SAN, were shown to synthesize fibronectin and laminin (detected on their cell surface and secreted to media). Collagen type VI was detected in cell media of CHP-126, IMR-32, and SH-SY5Y, and on the cell membrane of CHP-126, SH-SY5Y cells. It should be stressed that interstitial collagens (types I, III, and V) were not detected in the cell cultures [39].

### 2.2. ECM Content and Its Structural Features Can Aid NB Prognosis

Recently, collagen expression profiles in sections collected from intratumoral regions (IT), sites of tumor-stroma boundary (TS), and peripheral stroma (PS) of patient NB tumors were reported [40]. Thus, four different “collagen signatures” were evident: TS-high (COL6A1, COL10A1, COL18A1, and COL11A1); PS- and TS-high (COL17A1, COL11A2, COL28A1, COL2A2, COL2A1, COL16A1, and COL4A4); rich in all three regions investigated (COL12A1 and COL26A1), and PS-high (COL8A1, COL4A2, COL24A1, COL13A1, COL7A1, COL5A1, COL1A1, COL3A1, COL5A2, COL3A2, COL4A1, and COL1A2) [40]. Interestingly, COL11A1 (collagen type XI alpha 1 chain) was shown to be highly expressed by CAFs and tumor cells in patient samples. Co-culture of CAFs with SH-SY5Y NB cells enhanced tumor cell invasion, and CAF-associated COL11A1 expression was pivotal for the observed effect. Moreover, high TS COL11A1 staining in NB tumor samples was correlated with factors associated with poor prognosis and relapse. Additionally, low levels of COL11A1 mRNA were associated with the good survival probability [40].

Qualitative and quantitative changes of ECM components of the tumor stroma, such as fibrillar collagens and PGs, affect the architecture and biomechanical properties of the matrix, and can yield a stiff environment that supports the malignant cell phenotype, impairs drug delivery, and alters signaling pathways (e.g., by integrin clustering) to drive metastasis and chemoresistance (for a review on the topic, see [41]). With the application of digital image analyses and in large sets of NB tumor samples, several features of TME were analyzed to find correlates with prognostic factors used for NB risk stratification. Thus, the deposition of chaotic networks of highly cross-linked reticulin fibers on larger areas, along with a decrease in collagen type I deposition, resulted in a stiffer environment in samples of NB patients with a poor prognosis [42]. Moreover, irregularly shaped blood vessels and highly cross-linked reticulin fibers were associated with the ultra-HR NB disease [43]. Features of vitronectin “architecture”, i.e., high intensity of staining with pericellular and intracellular localizations vs. low and intercellular staining, were shown by Burgos-Panadero et al. to be valuable parameters of poor vs. favorable outcome stratification, respectively [38]. Additionally, the ECM protein was shown to be expressed by SK-N-SH and SK-N-BE(2) NB cells in a vitronectin KO mice model, stressing its tumor cell origin and contribution to the increased ECM stiffness [38]. Importantly, *MYCN*-amplified SK-N-BE(2) NB cells were shown to adapt to changes in the ECM content in vivo, and when the stiffness of the ECM was modelled in vitro in prolonged cultures in stiff gelatin-based hydrogels. This involved a process of selection of genetic changes (e.g., on chromosome 9) [44].

### 2.3. ECM Components of NB Are Affected by Differentiation

Retinoic acids are often used to investigate changes in ECM proteins and their receptors during NB differentiation. The differentiation of NB cell lines can yield cell populations with mixed composition of neurons, SCs, and even melanocytes, thus “mimicking” NCC maturation. For example, CHP-126 cells treated with all-trans retinoic acid (ATRA) yielded a major fraction of flat Schwannian cells, which exhibited a marked increase in the production of fibronectin, laminin, and collagen type IV, as well as small groups of neuronal cells with a decreased synthesis of the aforementioned ECM proteins [45].

Recently, Halakos et al. performed mass spectrometry (MS) analyses to identify changes in the proteome of SK-N-SH NB cells treated for 3 days with 13-*cis* RA [46]. The authors reported increased levels of differentiation-associated proteins, such as CRABP2, NEFM, NEFL, ICAM1, and PLAT, but also the upregulation of talin and paxillin. Interestingly, the “ECM organization” group was gathering the reduced proteins [e.g., the fibrillar collagens COL1A1, COL3A1, COL5A1, fibronectin (FN1)] [46]. Based on the data, it was concluded that the observed protein changes support a reduction in the ECM tension and the creation of a protein network that facilitates outgrowth of neurites.

In contrast, Tan et al. showed the increased expression of the *FN1* gene on mRNA and protein levels in ATRA-treated NB cells [47]. Additionally, *FN1* expression was positively correlated with outcomes of NB patients (see [47] for detail). The RNA sequencing analysis of ATRA-treated NGP NB cells revealed that the most enriched pathway was “ECM-receptor interactions” and pointed to the central position of FN1 in the “ATRA-affected interactome”. Moreover, siRNA-mediated FN1 downregulation had no effect on proliferation, but increased the migration and invasion of NGP and SH-SY5Y NB cells [47].

## 3. Proteoglycans Are Regulating Phenotype of NB Cells

Proteoglycans are universally expressed components of the ECM. Negatively charged sugar chain(s) of glycosaminoglycans (GAGs), which are linear and build of repeating disaccharide units, are linked to a protein core of PGs. The chains of GAGs of PGs are chondroitin sulfate (CS), dermatan sulfate (DC), heparan (Hep), heparan sulfate (HS), and keratan sulphate (KS) [35]. Roles of PGs in cancer are investigated: in the organization of ECM structural components (e.g., fibrillar collagens), in mechano-transduction, and as tissue hydrating factors. Importantly, PGs bind GFs, cytokines, and enzymes remodeling matrix components. As a result, PGs co-participate in the signaling of, e.g., membrane-bound receptor tyrosine kinases (RTKs) and Toll-like receptors (TLRs) during cell growth, survival, migration, and inflammation and angiogenesis [48]. Selected PGs are subsequently reviewed in the context of NB.

### 3.1. Small Leucine Rich Repeat Proteoglycans in NB

Biglycan (BGN), lumican (LUM), and decorin (DCN) are members of the family of small leucine rich repeat proteoglycans (SLRPs) [49]. Roles of BGN in the response to oxidative stress were investigated using sodium nitroprusside (SNP) as a donor of NO. The agent was shown to decrease levels of BGN in SH-EP1 and SH-SY5Y NB cells [50,51]. An anti-apoptotic role of over-expressed BGN in SNP-treated SH-EP1 cells was reported and linked to a downregulation of signaling from activated ERK1/2 and P38 [50]. In SH-SY5Y cells, over-expressed BGN exhibited a cell death protective effect that was correlated with an inhibition of autophagy, a decrease in AMPK signaling, and a reduction in levels of reactive oxygen species induced with NO [51].

DCN-positive tumor staining was detected in about 13% of NB patient samples and correlated with age ≤18 months, with INSS stages 1–3, and a lack of *MYCN*-amplification [52]. High levels of mRNA-encoding LUM in primary NB tumors were associated with a lower probability for overall survival (see [53] for more detail). Interestingly, DCN and LUM were strongly upregulated, on mRNA and protein levels, in floating spheroid cultures of SK-N-SH NB cells grown in serum-free (SF) neural stem cell medium to allow for enrichment of population of cancer stem cell-like cells (CSCLC), as compared with adherent cells [54]. More importantly, the secondary neurospheres generated from the cells positive for the PGs exhibited slow proliferation rates in a soft agar growth assay and a resistance to high-concentration temozolomide (>750 μM) [54]. Hence, DCN and LUM may affect cell plasticity, enable the generation of CSCLCs exhibiting the “quiescent phenotype”, as well as therapy resistance in conditions preventing cell attachment.

Finally, Salcher et al. showed that IMR-32, SK-N-SH, and SH-EP NB cells that ectopically over-expressed a transcription factor (TF) forkhead box O3 (FOXO3) upregulated LUM expression. Additionally, migration stimulation by LUM was reported in IMR-32, SK-N-SH cells over-expressing FOXO3 [53]. Finally, repaglinide—an insulin secretagogue—was shown to act as a FOXO3 inhibitor. Thus, it abolished FOXO3-medaited activity of promoters of *MMP13* and *MMP9*, prevented binding of the TF to repress *LUM* expression, and negatively affected the FOXO3-regulated migration of NB cells [53].

### 3.2. Roles of Neurocan in NB

Neurocan (NCAN) is a protein belonging to the lectican family of extracellular PGs [55]. Su et al. investigated roles of NCAN in NB. The authors reported that high expression levels of mRNA of NCAN correlated with poor overall survival (see [56] for detail). Additionally, the NCAN staining signal was stronger in HR NB tumors. In the model of TH-MYCN mice, NCAN was secreted to the ECM by neuroblasts in murine tumors [56]. Moreover, the ectopic over-expression of NCAN, e.g., in NB39 NB cells (exhibiting a low endogenous level of NCAN) yielded non-adherent, proliferating spheroid cultures in vitro, and enhanced aggressiveness of the cells in vivo. In contrast, downregulation of NCAN in the TH-MYCN-mouse-derived tumor cells impaired their proliferation and abolished tumorigenic potential. Finally, the treatment of NB39 cells with media from cells secreting NCAN resulted in changes of gene expression. Thus, it was reported that *CDKN1B* and neuronal differentiation genes such as *NSE* and *GAP43* were downregulated, but *MYCC*, *OCT4*, *SOX2*, *KLF4*, *ABCG2*, and *LGR5* were upregulated [56]. The authors hypothesized that NCAN may be a pivotal component of the tumor stem cell niche and can be involved in maintenance of the undifferentiated phenotype of NB cells.

### 3.3. Cell Surface PGs

Glypicans (GPC1-6) are anchored via glycosylphosphatidylinositol (GPI) to the cell membrane and contain 2 to 5 chains of HS. Syndecans (SDC1-4) are type I transmembrane glycoproteins that contain CS/HS chains. Both GPCs and SDCs are major representants of the group of cell surface PGs. Betaglycan (BGCAN, alias TGFBR3, transforming growth factor beta receptor 3) that contains HS/CS is yet another member of this group [55]. Selected examples of PGs containing chains of HS (HS-PGs) are discussed below in the context of NB research.

#### 3.3.1. Glypicans

Functions of GPCs in the development of the nervous system are well described. Thus, Kurosawa and co-authors reported that GPC2 binds to a heparin-binding protein, midkine, to regulate the adhesion and neurite outgrowth of N2a NB cells [57]. Roles of GPCs in cancer are investigated [55,58]. Notably, a loss-of-function mutation of the gene encoding glypican-3 (GPC3) is linked to Simpson–Golabi–Behmel syndrome, an overgrowth syndrome linked to the X chromosome, accompanied by an increased incidence of embryonal tumors such as hepatoblastoma, NB, gonadoblastoma, Wilms tumor, and hepatocellular carcinoma [59]. In NB samples of patients, Chan et al. reported a lack of GPC3 expression in all 136 cases studied by IHC [60], while Shibui et al. reported a positive GPC3 staining only in 1 of 35 samples [61]. Interestingly, in three cases, high serum levels of the protein were detected by ELISA, even though the NB samples were negative for GPC3 by IHC [61].

Recently, GPC2 was evaluated as a potential target for the immunotherapy of NB patients. Hence, among transcripts encoding GPC1-6, the highest levels were reported for GPC2 mRNA, and in NB, GPC2 mRNA was shown to be differentially expressed when compared with normal tissues [62]. Importantly, the high *GPC2* gene expression was correlated with poor overall survival and with the genomic gain of 7q [62]. GPC2 protein was identified in most HR NB samples and localized in the cell membrane, while being restricted from normal tissues. Interestingly, the authors reported the identification of a tumor-associated *GPC2* mRNA variant [62]. Mechanistically, MYCN was shown to bind to the promoter of *GPC2* to drive its expression. GPC2 was shown to be necessary for the proliferation of NB cells, and it was proposed to be engaged in the WNT-signaling pathway [62].

Glycerophosphodiesterase GDE2 (approved symbol GDPD5, glycerophosphodiester phosphodiesterase domain containing 5) is a transmembrane enzyme encoded on 11q13 which is known for its involvement in neuronal differentiation, e.g., by GPI cleavage to release cell-membrane-associated RECK (reversion inducing cysteine rich protein with kazal motifs) [63] and GPC6 [64]. In NB, high levels of mRNA of *GDPD5* were correlated with a probability of favorable overall survival. Moreover, GDPD5 over-expression in NB cells promoted differentiation (also cooperating with RA), increased cell adhesion to ECM proteins, and decreased cell migration [64]. Interestingly, in cells exhibiting the downregulated expression of *GDPD5*, differentially expressed genes were not only enriched with those involved in “neuronal differentiation”, but also with genes regulating “cell adhesion” (such as *LAMA4*, *COL6A3*, *COL13A1*, *FN1*, *ITGA10*, and *ITGA11*), “extracellular matrix organization” and “transmembrane receptor activity” [64].

#### 3.3.2. Roles of HS-PGs in NB Differentiation

Knelson et al. reported decreased levels of TGFBR3 mRNA in NBs, as compared with benign tumors, whereas high TGFBR3 mRNA levels were correlated with EFS in NB patients (see [65] for detail). Moreover, levels of the TGFBR3 protein were decreased in late-stage NB tumors. Mechanistically, expression of the *TGFBR3* gene was shown to be repressed by MYCN [65]. The authors proposed that high expression levels of mRNAs of TGFBR3 and SOX10 could be biomarkers of a response to differentiation therapy [65].

Furthermore, levels of mRNA encoding for HS-PGs TGFBR3, GPC1, GPC3, SDC3, and SDC4 were significantly lower in stoma-poor NB tumors, as compared with stroma-rich NBs. The protein staining was stroma-associated with Schwan cells and decreased in late-stage NB tumors. Moreover, patients with high levels of three mRNAs encoding TGFBR3, GPC1, and SDC3 had higher EFS [66].

Additionally, stimulating effects on NB differentiation, i.e., neurite outgrowth and the expression of differentiation markers, recombinant soluble TGFBR3 [65], GPC1, GPC3, SDC3, and SDC4, and the transient over-expression of soluble TGFBR3, GPC1, GPC3, and soluble heparin were reported [66]. NB differentiation was dependent on the HS modification of TGFBR3 and abrogated by blocking TGFBR3 shedding [66]. Mechanistically, HS-PGs and heparin-transduced signals via the phosphorylation of FGFR1 and ERK to increase the TF ID1 [65,66].

**Figure 1:**
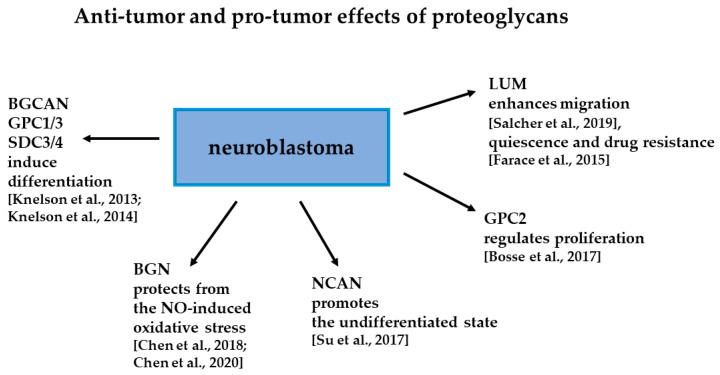
Anti-tumor and pro-tumor effects of selected proteoglycans in NB (see Section 3 for more detail) [50,51,53,54,56,62,65,66].

Binding GFs to their receptors can be modified by interactions with HS-PGs. Thus, TGFBR3 was shown to bind to FGF2 and FGFR1 in a GAG-dependent manner [65]. FGF2 alone and in combination with soluble TGFBR3, SDC3, and SDC4 led to an increase in differentiation markers [66]. Finally, in NB patient tumors, FGF2, IGF1, and heparin-binding EGF were among the most differentially expressed GFs in stroma-rich tumors, as compared with stroma-poor samples. The high serum level of FGF2 was associated with the higher EFS. Thus, the detection of serum levels of FGF2 was proposed as a prognostic and differentiation biomarker in NB [66]. Figure 1 summarizes the discussed roles of PGs in NB.

### 3.4. Enzymes Modifying HS-PGs

Post-translational modifications add to the structural diversity of PGs and can modulate their functions [35,55]. Knelson et al. reported that high expression levels of mRNA of sulfotransferases, i.e., heparan sulfate 2-O-sulfotransferase 1 (HS2ST1), heparan sulfate 6-O-sulfotransferase 2 (HS6ST2), heparan sulfate 6-O-sulfotransferase 3 (HS6ST3), and N-deacetylase and N-sulfotransferase 2 (NDST2), were correlated with a good prognosis in NB. In contrast, high expression levels of mRNA of sulfatases 1 and 2 (SULF1 and SULF2) were associated with a poor prognosis [66].

Heparinase (HPSE) is a glycoside hydrolase that cuts off HS polysaccharides from HS-PGs to participate in ECM turnover, and the release of GFs, cytokines, chemokines, and enzymes. HPSE plays roles in both tissue homeostasis and pathological states [35]. In the TME, the activity of HPSE affects phenotypes of tumor and stromal cells (such as fibroblasts, macrophages, and endothelial and NK cells) regulating their proliferation, migration, invasion, and processes of angiogenesis and inflammation. Interestingly, HPSE non-enzymatic roles were also described, e.g., the regulation of cell signaling and gene expression [67].

Zheng et al. reported that HPSE was detected in NB cells in about 62% of tumors (by IHC), mostly in their cytoplasm, but a lack of a stromal signal was revealed. The expression of heparinase was associated with poor outcomes [68].

Qu et al. reported a positive association between levels of miR-558 and mRNA of HPSE (or heparinase protein levels) in poorly differentiated NBs [69]. Mechanistically, miR-558 was shown to bind to the promoter of the *HPSE* gene to activate its transcription, resulting in an increase in HSPE (and indirectly VEGF) to enhance the viability, invasion, and migration of NB cells, as well as the tube formation of endothelial HUVEC cells. Moreover, the downregulation of miR-558 in mice impaired tumor growth after the s.c. injection of SH-SY5Y cells, as well as lung metastasis and angiogenesis [69]. Finally, it was reported that the SMAD4 protein and the *HPSE* expression levels were inversely correlated in NB tumors, and low SMAD4 mRNA levels were associated with the lower probability of survival [70]. Additionally, SMAD4 was shown to bind to the promoter of the *HPSE* gene to repress its LEF1-mediated expression in NB cells to impair their viability, invasion, metastasis, and angiogenesis (in vitro and in vivo) [70].

## 4. Overview of Selected NB Receptors Engaged in Interactions with the ECM

Cells communicate with ECM components, and the major group of receptors engaged in the process is an integrin. Other transmembrane or membrane-associated proteins, such as Ig-like cell adhesion molecules, neogenin 1 (NEO1), ribosomal protein SA, or even glycolipids, can be involved, which will be discussed below in the context of NB.

### 4.1. Integrins Are Major Adhesion Receptors Which Link the Cytoskeleton to ECM

Integrins are type I transmembrane adhesion receptors responsible for bi-directional signal exchange between the ECM and the actin cytoskeleton. There are 24 possible combinations of integrin heterodimers, built of α (1 of 18 subunits) and β (1 of 8 subunits) chains. Integrins are key players in responding to ECM components, but also regulate the turnover of ECM proteins [71]. Integrins receive signals from both outside and inside of cells, which results in changes in their activation and further signal transduction. On the cell outside, integrins interact with a plethora of matrix proteins, depending on the heterodimer composition, and some can be “promiscuous” [71]. Integrins, with exception to the β4 subunit that contains the large cytoplasmic domain, do not directly transduce signal. Rather, via their cytoplasmic tails, they associate with other adaptor proteins of focal adhesions, such as α-actinin, talin, tensin, paxillin, ezrin, FAK, and SRC [72]. Roles of the receptors as “integrators of ECM-cell cytoskeleton communication” in adhesion, cell spreading, migration are well established in both normal processes such as development, tissue homeostasis, but also in pathogenesis, including cancer [73,74]. Moreover, integrins can also engage binding partners in the cell membrane (e.g., RTKs, UPAR, and glycolipids), which adds complexity to their network of contacts. This can affect cell fate—cell survival or death—as well as stemness, differentiation, and resistance to drugs [71,73].

#### 4.1.1. Integrin Expression in NB

The expression of integrins was analyzed in patient samples to show the uniform presence of integrin β1 [75,76] and αv [76], irrespective of the NB differentiation stage, and also in the adrenal gland medulla (in pheochromocytes). The subunits α1 and α3 were also present in the majority of samples analyzed [75]. However, the β3 subunit was shown to be expressed in undifferentiated, and to some extent, differentiating NB tumors (in neuroblasts and ganglion cells), and was absent in GNB and the adrenal gland medulla samples. The β5 integrin subunit was not detected in NB tumors and the adrenal medulla, but was consistently present only in ganglion cells of GNB samples [76].

#### 4.1.2. Integrins Play Roles in NB Differentiation

Interactions between ECM and integrins on NB cells accompany phenotypic changes, such as neurite elongation, induced during differentiation. Several compounds can be used for differentiating NB cells. Thus, ATRA-treated SK-N-SH cells exhibited elongated neuritic processes on vitronectin and laminin coated-surfaces in a process mediated by the β1 integrin subunit [37]. Additionally, ATRA-treated SH-SY5Y cells exhibited neurite extension and longer neurites on laminin, which correlated with upregulation of the integrin heterodimer α1β1. In both undifferentiated and differentiated SH-SY5Y cells, the extension of neurites on laminin could be blocked by anti-β1 antibodies (Ab), leading to cell detachment [77]. Similarly, in LA-N-5 NB cells, RA increased the α1 and β1 subunits, and INFγ (alone or combined with TNF-α) increased the α1, α2, α3, and β1 subunits [78].

#### 4.1.3. Integrins Play Roles in NB Survival

Roles of integrins in cell death regulation of NB cells were also reported [79,80]. Apoptosis was induced in ATRA-differentiated SK-N-BE cells grown in suspension in a SF medium, as compared with undifferentiated cells. In the model, the cells could be rescued from the cell death by, e.g., cell attachment to plates coated with collagen type I, laminin, anti-β1 antibody, but also for cells in suspension by soluble collagen type I and complexing integrins with Abs against the α1, β1, β3, α3, αν subunits, and rabbit anti-mouse Ig Abs [79]. Similarly, anti-β1 mAb caused apoptosis in attached LA-N-5 cells differentiated with RA, which was related to the downregulation of AKT and the cleavage of FAK [80].

#### 4.1.4. Roles of Integrins in Metastasis of NB Cells

Integrins can regulate the malignancy of tumor cells by affecting steps of the metastatic cascade [81,82]. Through analysis of microarray expression data, Young et al. proposed that integrin α4 could be associated with the poor prognosis of patients with NB that lack the *MYCN* amplification [81]. The authors showed that NB5 NB cells ectopically expressing the subunit α4 exhibited enhanced adhesion on and migration toward the connecting segment 1 (CS1) of fibronectin or VCAM in vitro. Additionally, after i.v. injection, NB cells over-expressing the α4 subunit produced more macroscopic metastases in the liver [81].

Roles of integrins in the degradation of ECM components have been reported. In SK-N-SH cells, mainly expressing the integrin α3β1 heterodimer, the receptor mediates binding of the cells to collagen type IV. Moreover, in cells grown on collagen-IV-coated surfaces, the levels of the α3 and β1 subunits were decreased, but increased levels of activated MMP2 and MMP9, as well as TIMP1 and TIMP2, were measured, as compared with cells grown on plastic. The application of combined anti-α3 and anti-β1 mAbs to cells grown on collagen-type-IV-coated surfaces resulted in higher levels of secreted MMP2 [82].

#### 4.1.5. FAK Is Upregulated in MYCN-Amplified NB

Focal adhesion kinase (FAK, now officially named protein tyrosine kinase 2, PTK2) is a cytosolic tyrosine kinase and a key component of the focal adhesion complex to regulate cell motility [72]. STRAP (serine–threonine kinase receptor associate protein), a scaffold protein, was recently shown to regulate FAK activity and was associated with a poor outcome in NB [83]. Beierle et al. reported increased FAK staining in *MYCN*-amplified NB tumors from patients with INSS stage 4 disease [84]. Higher levels of FAK were detected in NB cells over-expressing MYCN. Mechanistically, MYCN was shown to bind to the promoter of the FAK-encoding gene to activate its expression [85]. The downregulation of FAK expression with siRNA in *MYCN*-amplified SK-N-BE(2) NB cells was shown to reduce invasion and migration in vitro, in a more evident way, as compared with *MYCN* non-amplified SK-N-AS NB cells [86]. FAK is a target for the development of anti-cancer therapies (see Section 7).

#### 4.1.6. Roles of MDA-9/Syntenin 1 in NB

Recently, Bhoopathi et al. characterized the function of melanoma differentiation-associated gene-9 (MDA-9) also known as syntenin 1 (syndecan binding protein, SCDBP, is now the officially approved name of the protein) in NB [87]. MDA-9/syntenin 1 is a scaffold protein with one N-terminal domain, two tandem PDZ domains, and one C-terminal domain. It is over-expressed in several cancers and shown to interact with, e.g., EGFR, IGF1R, FAK, and SRC to enhance metastasis (for a recent review, see [88]). Thus, levels of syntenin 1 mRNA were elevated in bone marrow NB samples of patients, as compared with primary tumors. Additionally, the shRNA-mediated downregulation of synenin 1 or inhibition of the PDZ1 domain of the protein with a specific antagonist (PDZ1i) impaired migration and invasion of SK-N-AS, SK-N-SH, and NB1691 NB cells. Mechanistically, both approaches were shown to downregulate levels of the integrin subunits α6 and β4, MMP9, MMP2, cofilin, N-cadherin, vimentin, and activated SRC, RHO A, RAC1, CDC42 proteins, but upregulate levels of RECK in vitro. Finally, both approaches decreased metastatic tumor growth in vivo to extend the survival of animals, stressing that the PDZ1 antagonist may further optimized to develop an anti-cancer drug candidate [87].

#### 4.1.7. Roles of Caspase 8 in NB

Integrins in cooperation with apoptosis initiator caspase 8 (CASP8) can regulate cell fate based on clues from TME. As mentioned above, unbound integrins can transduce signals to induce apoptosis (see [79] and the discussion below). However, cancer cells evolve ways to escape programmed cell death. A lack of CASP8 expression due to the methylation of the *CASP8* gene is a common feature of NB tumors and is associated with a poor prognosis [89]. Teitz et al. reported that a loss of CASP8 expression in NB7 NB cells with ectopic expression of the protein was a pivotal step allowing for metastatic growth in mice [90]. Additionally, CASP8-positive cells expressing the α3β1 integrin heterodimer (binding laminin, but not collagen) died in a 3D collagen matrix, whereas increased survival was observed for cells with low levels of the α3β1 integrin. A concomitant lack of expression of the α3β1 integrin and CASP8 was correlated with the maximal cell survival [90].

Interestingly, CASP8 may also play non-apoptotic roles. Thus, after integrin ligation, CASP8 is recruited to the focal adhesion complex, and interacts with FAK and carboxypeptidase N subunit 2 (CPN2) to regulate cell migration and CPN2 activity, independently of its enzymatic activity [91].

Finally, to gain more insight into roles of CASP8 in metastasis, an NB model over-expressing MYCN and a lack of CASP8 was introduced based on the TH-MYCN mice [92]. A characteristic feature of the model was an increase in bone marrow metastasis, as compared with animals over-expressing MYCN with wild-type levels of CASP8 (although primary tumor formation was unaffected). Analyses of transcriptome changes in primary tumors in both models revealed the differential expression of genes involved in metastasis, EMT, fibrosis, extracellular matrix detachment, wound healing, and inflammation. The “ECM structural constituents” was the most enriched category of differentially expressed genes (including upregulated *TFPI2*, *LAMA4*, *FBLN2*, *PRELP*, and *COL4A2*) [92].

### 4.2. Laminin Receptor/Ribosomal Protein SA

Laminin is a basement membrane component and yet another ECM glycoprotein involved in regulation of cancer cell phenotype. It is bound by, e.g., the α3β1, α6β1, α7β1, and α6β4 integrin heterodimers [93], as well as by the laminin receptor/ribosomal protein SA (RPSA, also known as LRP). The receptor is a membrane-associated glycoprotein with multiple functions, extending beyond laminin binding, i.e., cytoskeletal, ribosomal, and nuclear [94]. Interestingly, ATRA was shown to decrease the level of the 67 kDa LRP, which was associated with a reduction in SH-SY5Y cell migration in vitro. Additionally, in HR NB tumors, the receptor was expressed in 71% of cases, as shown by IHC [95]. In IMR-32 NB cells, siRNA-mediated downregulation of expression of 37 kDa LRP reduced cell viability, proliferation, and caused apoptosis [96]. The receptor is a target for the development of anti-cancer therapies (see Section 7).

### 4.3. Ig-like Cell Adhesion Molecule Family

Members of the Ig-like cell adhesion molecule family play roles in homophilic and heterophilic interactions (the latter include other cellular receptors, such as integrins, as well as ECM components) to regulate cancer cell phenotype, including cell survival and collective cell migration [97]. Below, as examples, selected publications on the roles of members of the cell adhesion molecule (CAM) family in NB are discussed.

#### 4.3.1. Intercellular Adhesion Molecule 2

Intercellular adhesion molecule 2 (ICAM2) is a transmembrane glycoprotein shown to interact with actin via α-actinin to regulate ECM adhesion and cell motility [98]. Thus, the migration of ICAM2-over-expressing SK-N-AS cells on collagen type I was still possible, but it was almost completely reduced on vitronectin, and inhibited on fibronectin [98]. Yoon et al. reported association of the positive ICAM2 staining with a good prognosis and suggested its inverse correlation with a NB metastatic potential [99]. Mechanistically, when ICAM2 was ectopically over-expressed in SK-N-AS cells, it caused re-positioning of the actin fibers along with the cell membrane, and a drop in cell motility in vitro. Additionally, such cells failed to induce metastases after i.v. injection to mice, despite being capable of s.c. growth [99]. Importantly, Feduska et al. showed that N-linked glycosylation of ICAM2 is to some extent necessary to confer metastasis-suppressive features of the receptor [100].

#### 4.3.2. Neural Cell Adhesion Molecule 1

Neural cell adhesion molecule 1 (NCAM, CD56, located on 11q23) was expressed in the majority of NB samples of a tissue microarray (also in the brain and adrenal tissues), even in NB samples with the 11q23 deletion. However, expression levels of mRNA encoding the NCAM 120 and 180 kDa isoforms were significantly associated with GNB and NB tumors, respectively. Western blot analysis of protein samples of NB tumors showed the presence of the signal of the 180 kDa, but not the 120 kDa band, whereas GNB tumors predominantly showed the presence of the band of 120 kDa and a weak signal of the 180 kDa band. No statistical significance was found for the 140 kDa isoform between the groups investigated [101].

#### 4.3.3. Cell Adhesion Molecule L1-Like

The low expression of mRNA encoding cell adhesion molecule L1-like (CHL1) was associated with a decreased survival and an NB relapse [102]. In IMR-32 cells, CHL1 over-expression increased levels of the differentiation marker—microtubule associated protein 2 (MAP2), upregulated autophagy, apoptosis, inhibited cell growth, and anchorage-independent colony formation, reduced the activation of RAC and CDC42, decreased the phosphorylation of P38/JNK, AKT, impaired migration and invasion, and reduced orthotopic tumor growth in mice. Opposite effects were reported for HTLA-230 NB cells with the shRNA-mediated downregulation of CHL1 [102]. Recently, ezrin was reported to bind to CHL1 and to mediate the receptor-suppressive function [103].

#### 4.3.4. L1 Cell Adhesion Molecule

Zaatiti et al. reported that L1 cell adhesion molecule (L1CAM) was among 10 most highly over-expressed proteins in *MYCN*-amplified IMR-32 cells, as compared with *MYCN* non-amplified SK-N-SH cells [104]. The *L1CAM* mRNA and protein levels were increased in NB samples, as compared with normal tissues [105]. The expression of L1CAM mRNA was inversely correlated with levels of miR-375, and the latter was shown to regulate the L1CAM-encoding gene expression, also affecting proliferation and radiosensitivity. Additionally, lncRNA XIST was shown to sponge miR-375 to regulate L1CAM expression [105].

Finally, siRNA-mediated L1CAM downregulation in IMR-32 cells impaired cell proliferation and migration, as well as the growth of tumorspheres. Additionally, after a single dose of radiation, L1CAM and MYCN were upregulated in the cells, whereas L1CAM knockdown decreased MYCN, increased PTEN levels, and radio-sensitized IMR-32 cells [106].

### 4.4. Roles of CD44 in Regulation of Phenotype of NB Cells

CD44 molecule (Indian blood group), CD44, is a transmembrane glycoprotein and a hyaluronan (HA) receptor that participates in cell–cell and cell–ECM interactions [35]. CD44 can be expressed in several isoforms depending on the alternative splicing of mRNA of the *CD44* gene that contains 19 exons. Additionally, the protein can be post-translationally modified by N-, O-glycosylation, phosphorylation, and the addition of GAGs [35].

In the context of NB, a lack of expression of CD44 in advanced stages of the disease was reported [107,108] and it was associated with *MYCN*-amplification [108]. In SK-N-SH and SK-N-BE cells, only the CD44s (s refers to standard) isoform was expressed and upregulated with differentiating agents [108]. Additionally, in SH-EP cells expressing CD44s, tunicamycin (an inhibitor of N-glycosylation) impaired binding to HA [109]. Fichter et al. reported that PDGF and IGF1 upregulated CD44s and CD44v6 proteins on the SK-N-SH cell surface, which correlated with enhanced binding to HA [110].

Several groups characterized phenome features of CD44s-negative and -positive subpopulations of NB cells, including their tumorigenicity. Thus, Siapati et al. reported that CD44s-positive cells were flat and proliferated faster, while CD44s-negative exhibited neuronal projections, proliferated more slowly, and adhered less, which was correlated with the lower expression of α4, α5, β1 integrins, and ICAM. Moreover, after the s.c. injection of CD44s-negative cells to mice, tumors developed at the injection site, and the cells selectively metastasized to the spleen, and yielded a higher percentage of animals with bone marrow and liver metastases [111]. In contrast to the aforementioned results, Valentiner et al. reported a higher tumorigenicity of CD44-positive NB cells [112]. Vega et al. showed that CD44-positive SK-N-SH cells were more adherent and motile, and proliferated faster (both in 2D and 3D cultures) as compared with CD44-negative cells. Additionally, the authors characterized CD44-positive cells of SK-N-SH tumorspheres, and reported the presence of a small fraction of “neural-crest like” multipotent cells that yielded neuronal, glial, and mesenchymal lineages in appropriate differentiation conditions [113]. Additionally, CD44-positve cells, sorted out of the primary mice xenografts, were more aggressive to yield primary tumors with an increased incidence, but also more metastases to lungs [113]. Finally, Ognibene and Pezzolo showed that neurospheres derived from IMR-32, ACN and SH-SY5Y cells expressed CD44v6, along with CD114, NCL, NPM1, PES1, and GPC2 [114]. Interestingly, CD44v6 was detected in rare populations of NB cells of the INSS stage 4 tumors before chemotherapy, and the percentage of cells increased after chemotherapy [114].

### 4.5. Dependence Receptors

Dependence receptors (DRs) induce cell death in the absence of a stimulus (when unbound by a ligand). DRs are transmembrane proteins forming a group of 20 members, including NGFR, TRKA, TRKC, MET, RET, ALK, DCC, NEO1, IR, IGF1R, and some integrins [115]. Their roles in cell death induction have also been investigated in cancer [116]. As examples of DRs relevant to the topic of this review, DCC netrin 1 receptor (DCC, previously named deleted in colorectal carcinoma) and its homologue neogenin (NEO1) are discussed. Both receptors bind to netrin, which is a laminin-related protein [117].

DCC was shown to play a role in neurite outgrowth, axon guidance, and migration of NCCs. DCC and netrin 1 (NTN1) were pivotal to induce neurite outgrowth by the activation of RAC1 and CDC42 in mouse N1E-115 NB cells, but the downregulation of RHO A and RHO A kinase [118].

In primary NB tumors, Kong et al. analyzed polymorphism of the *DCC* gene at codon 201, in which the C->G transversion resulted in the missense mutation of CGA (encoding Arg) to GGA (encoding Gly) (in one or two alleles in the gene) [119]. The most common codon 201 was GGA, which correlated with a NB metastatic disease, then CGA/GGA, followed by CGA. The relationship between 201 Gly and a lack of the DCC protein remains to be established [119].

NEO1 was shown to be expressed in NB tumors; mostly, tumor-cell-associated staining was detected. The paracrine signaling of NTN1 via NEO1 was analyzed in SK-N-SH cells, with the low endogenous expression of NTN1 [120]. Hence, the siRNA-mediated downregulation of NEO1 in SK-N-SH cells impaired transwell migration, as well as the migration on fibronectin of spheroid-derived cells. After s.c. injection of the cells to mice, primary tumor growth was unaffected; instead, NEO1 downregulation impaired spontaneous spreading to the spleen, kidneys, liver (the cells were found exclusively in lungs). Mechanistically, NEO1 was reported to complex NTN1, integrin β1, and FAK. NTN1 and NEO1 signaling led to activation of the integrin β1 subunit and the phosphorylation of FAK [120].

NTN1 interacts with DCC and UNC5H proteins (unc-5 netrin receptor, UNC5HA-D, previously UNC5H1-4). Delloye-Bourgeois et al. analyzed the expression of netrin 1 and its receptors DCC and UNC5H1-4 in metastatic NBs [121]. The authors reported that the INSS stage 4 tumors of patients >1 year of age exhibited higher levels of NTN1 mRNA and NTN1 protein (as compared with patients of stage 4S or stage 4 <1 year of age). NTN1 was almost exclusively tumor-cell-associated. Additionally, DCC was weakly expressed, whereas UNC5H1-4 expression patterns were non-discriminative between the analyzed groups [121]. In IMR-32 cells, NTN1 membrane staining was detected; however, the protein was secreted, and its decrease resulted in apoptosis [121].

Finally, SK-N-SH cells were reported to express NTN4 and NEO1, and both proteins were shown to associate with each other [122]. NTN4 promoted the survival of NEO1-positive cells and acted as a chemoattractant to regulate their migration on fibronectin in vitro. Furthermore, NTN4 downregulation blocked lung metastases in a chicken embryo model [122]. Additionally, NTN4 was reported to be strongly expressed in NB tumors, i.e., in tumor cells, stroma, and blood vessels. In the context of NB cells and ECM interactions, a complex of laminin γ1 and NEO1 was immunoprecipitated from SK-N-SH cells [123].

## 5. Glycocalyx Changes Affect Interactions of NB Cells with the ECM

Proteoglycans, glycoproteins, and glycolipids are the major groups of glycocalyx. Changes in glycosylation are universally observed in cancer and affect phenotypes, including proliferation, survival, adhesion, and migration of tumor cells [124]. It is now well established that glycocalyx-mediated interactions of tumor cells with the TME can impact therapy response. Importantly, such interactions are exploited to target cancer cells [125].

### 5.1. Roles of Glycosylating Enzymes in Regulation of NB Aggressiveness

Several reports have analyzed the effects of protein glycosylating enzymes on phenotypes of NB cells and their ECM interactions to show that they can mediate both pro- and anti-tumor effects.

Inamori et al. reported that higher levels of mRNA of GnT-V (MGAT5, alpha-1,6-mannosylglycoprotein 6-beta-N-acetylglucosaminyltransferase) were measured in INSS stages 1, 2, and 4S, as compared with stages 3–4. Additionally, siRNA-mediated MGAT5 downregulation impaired ATRA-induced apoptosis in CHP-134 NB cells [126]. Hall et al. showed that knockout of the gene *MGAT1* (alpha-1,3-mannosyl-glycoprotein 2-beta-N-acetylglucosaminyltransferase, alias GNT-1) in rat NB cells led to changes in cell morphology, growth, adhesion, and invasion [127]. Abbott et al. analyzed effects of GnT-Vb (MGAT5B, alpha-1,6-mannosylglycoprotein 6-beta-N-acetylglucosaminyltransferase B) or POMGNT1 [O-linked mannose N-acetylglucosaminyltransferase 1 (beta 1,2-)] on the migration of SH-SY5Y cells [128]. Ectopic over-expression of GnT-Vb in SH-SY5Y enhanced ATRA-stimulated neurite outgrowth on laminin. Moreover, shRNA-mediated GnT-IX and POMGNT1 downregulation enhanced spreading, which could be abolished with anti-β1 integrin Ab, decreased motility, and impaired ATRA-stimulated neurite outgrowth (all on laminin). Finally, GnT-Vb knockdown in SH-SY5Y cells resulted in a high membrane content of the integrin β1 subunit [128]. Hsu et al. showed that positive staining of B4GALNT3 (beta-1,4-N-acetyl-galactosaminyltransferase 3) predicted a favorable outcome [129]. Mechanistically, B4GALNT3 was upregulated in ATRA-treated SK-N-SH cells. Ectopic over-expression of B4GALNT3 in the cells reduced proliferation and impaired migration and invasion. Additionally, β1 integrin was modified by B4GALNT3, leading to the decreased phosphorylation of FAK, SRC, paxillin, AKT, and ERK1/2 [129].

Effects of beta 3-glycosyltransferases and beta 4-glycosyltransferases on malignant properties of NB cells were also analyzed. Thus, Ho et al. reported that the positive IHC-staining of B3GNT3 (UDP-GlcNAc:betaGal beta-1,3-N-acetylglucosaminyltransferase 3) predicted 5-year overall survival. The ectopic over-expression of B3GNT3 in SK-N-SH cells reduced colony formation in soft agar, migration, and invasion, and decreased the phosphorylation of FAK, SRC, paxillin, AKT, and ERK1/2 [130]. Finally, Chang et al. reported that upregulated IHC-staining of B4GALT3 (beta-1,4-galactosyltransferase 3 beta4Gal-T3) predicted a lower probability for 5-year overall survival in NB patients [131]. In NB models, B4GALT3 over-expression in SH-SY5Y cells enhanced tumor growth in vivo (after s.c. injection), yielding tumors with poorer stroma, but higher blood vessel density. Additionally, it enhanced β1-integrin-mediated migration, as well as invasion in vitro. Interestingly, B4GALT3 was shown to modify β1 integrin, and increased the level of its mature form in SH-SY5Y cells. The phosphorylation of FAK was increased in SH-SY5Y cells over-expressing B4GALT3, grown on fibronectin or laminin [131].

### 5.2. Ganglioside GD2

Gangliosides are glycosphingolipids enriched in nerve tissues and over-expressed in neuroectodermal tumors [132]. Tumor-associated carbohydrate antigens (TACAs) are accumulated in the outer cell membrane, anchored via ceramide, while the linear or branched carbohydrate chain (with one or more sialic acid (Sia) molecules) is exposed to the cell outside [133]. Modifications of Sia can yield O-acetyl- or N-glycolyl-gangliosides that are tumor-specific epitopes and can be targeted with antibodies [134,135]. Moreover, gangliosides are components of lipid rafts and can modulate the signaling of RTKs [136].

GD2 ganglioside (GD2) is over-expressed on NB [137], and its oligosaccharide part consists of glucose, galactose, N-acetylgalactosamine, and two Sia molecules. GD2 is used for NB diagnosis [138] and it is a target of approved NB immunotherapy with application of anti-GD2 mAbs [137]. GD2 ganglioside is known to regulate NB signaling, and anti-GD2 mAbs are used to investigate this phenomenon (for a review, see [139]). Thus, anti-GD2 mAb 14G2a cause the aggregation and detachment of NB cell lines, e.g., IMR-32, LA-N-1, and decreased cellular ATP levels [140]. The observed cytotoxicity was correlated with the decreased activity of aurora proteins, nuclear localization of P53, AKT/mTOR downregulation, and the induction of apoptosis in IMR-32 cells [140,141,142]. Recently, ch14.18/CHO mAb (dinutuximab beta) was also shown to induce the cytotoxic cell death of IMR-32 and CHP-134 cells [143]. In the context of NB cells and ECM–protein interactions, 14G2a mAb reduced the attachment of IMR-32 cells to fibronectin [142]. Additionally, an inhibitor of integrin complexes αvβ3, αvβ5 (SB273005), potentiated the cytotoxic effects of 14G2a mAb on LA-N-5 and Kelly cells. Similarly, an inhibitor of the active integrin complex α4β1 (BIO1211) potentiated the cytotoxic effects of 14G2a mAb on IMR-32 cells [144].

Kazarian et al. reported that ganglioside depletion in LA-N-1 NB cells with D-threo-1-phenyl-2-decanoylamino-3-morpholino-1-propanol-HCl (D-PDMP) yielded cells with reduced attachment and migration on collagen type I, as well as invasion to Matrigel [145]. Importantly, GD2 is known for its immunosuppressive functions, can be shed from NB cells, and it is present in blood of NB patients [146]. GD2 was shown to activate integrin signaling by clustering α2β1 integrin on platelets [147] and affecting α2-integrin-mediated FAK activation [148]. Finally, Jabbar et al. reported that exogenously supplied GD2 increased the adhesion of platelets to collagen type I [149]. Thus, GD2 is also of primary research importance in the context of NB cells and TME interactions.

## 6. NB and ECM Degradation

Matrix metallopeptidases (MMPs, previously named matrix metalloproteinases) are endopeptidases containing zinc with multiple roles in the TME. MMPs digest structural ECM components to facilitate invasion through the basal lamina and stroma, but also release of GFs and cytokines. Remodeling of the ECM allows for the migration of tumor cells, but also endothelial cells, e.g., via the activity of matrix metallopeptidase 2 (MMP2) and matrix metallopeptidase 9 (MMP9) [150]. The activity of ECM remodeling enzymes and their inhibitors is balanced in homoeostasis and can be dysregulated in pathological conditions. MMPs can be produced by tumor cells and by accessory cells in the TME (e.g., fibroblasts, endothelial cells, and immune cells). Their roles in tumor progression and therapy resistance have also been investigated in the context of possible anti-cancer treatments [150].

### 6.1. MMPs and Their Inhibitors

Several lines of evidence support the roles of MMPs in aggressive features of NB. Thus, IHC analyses of patient NB samples showed that MMP2 and MMP9 were expressed together, mostly in the ECMs of tumor-adjacent stroma and endothelial cells, while TIMP2 (TIMP metallopeptidase inhibitor 2) was detected in neuroblastic tumor cells, their adjacent stromal tissue, and in endothelial cells. The presence of peritumoral MMP2 or the absence of TIMP2 were both associated with advanced NB stages and poor prognosis markers [151]. Additionally, Zhang et al. reported that the cytoplasmic and membrane-associated MMP14 (matrix metallopeptidase 14) staining of tumor cells in NB patient samples correlated with poor prognoses [152].

Interestingly, in 3D models of spheroids grown in gels of collagen type I, MMPs (including MMP2) mediated the incorporation of invasive CD44-positive, S-type SHEP cells between non-invasive CD44-negative, N-type IMR-32 cells to enable their cooperative migration [153]. Moreover, in a model of double-deficient RAG1 and MMP9 mice, the orthotopic or s.c. injection of SK-N-BE-(2).10 NB cells yielded tumors with smaller blood vessels with a decreased pericyte cell content [154]. Recently, Somasundaram et al. reported the roles of MMP9, produced by NB cell lines after treatment with radiotherapy, on the activation of NFκB-mediated transcriptional activity, which led to transcriptomic and phenotypic changes including the sustained production of MMP9 [155]. Finally, the regulation of MMPs expression involves miRNAs. Thus, Yuan et al. showed that miR-338-3p targets MMP2 mRNA [156], while Zhang et al. reported that miR-9 targets MMP14 mRNA in NB [152].

Roles of metallopeptidase inhibitors are also being elucidated. Thus, metallopeptidase inhibitor 1 (TIMP1) mRNA expression was correlated with a lower overall survival probability of NB patients [157]. Jaworski and Peréz-Marinéz reported that TIMP2 is upregulated during the differentiation of neuronal cell lines and NB cell lines [158]. Finally, TIMP3 (TIMP metallopeptidase inhibitor 3) over-expression, by the retroviral vector delivery to NXS2 NB cells, had anti-proliferative effects on the growth of tumor cells after s.c. injection to mice [159]. Xin et al. reported studies on RECK, which is a GPI-membrane-anchored glycoprotein and an inhibitor of MMP2, MMP9, and MMP14. Thus, miR-15a was shown to target mRNA of RECK, and levels of miR15a and mRNA of RECK were inversely correlated in NB samples. siRNA-mediated RECK downregulation increased the migration of SK-N-SH and GI-LA-N cells in vitro and enhanced the secretion of MMP9, but not MMP2 and MMP14 [160].

### 6.2. Plasminogen Activator (UPA) and Plasminogen Activator Receptor (UPAR)

In the TME, functions of a ligand PLAU (alias UPA, plasminogen activator, urokinase) and its receptor PLAUR (alias UPAR, plasminogen activator, urokinase receptor) comprise ECM remodeling and signaling between cells and ECM to regulate cell fate, adhesion, and motility, as well as angiogenesis and inflammation. UPAR is cell-membrane-anchored by GPI and can be released by GDE3 (glycerophosphodiester phosphodiesterase domain containing 2). Additionally, the receptor was shown to bind vitronectin and participate in the signaling of integrins and RTKs [161]. The UPA/UPAR system is responsible for direct digestion of the ECM, but also for the digestion of plasminogen to plasmin to activate MMPs. Hence, the system is a target for the development of anti-cancer therapies [162].

With reference to the expression profiles of components of the UPA/UPAR system in NB tumor samples, Li et al. reported mostly membrane-bound and cytoplasmic tumor cell signals of UPA and UPAR. Importantly, the high expression signals of UPA and UPAR were correlated with HR NB, and their concomitant positivity was an independent poor prognosis factor [163]. Additionally, Shmakova et al. reported that high mRNA levels of UPAR were related to poor overall survival, although no prognostic values were linked to the mRNA of UPA or mRNA of SERPINE1 (serpin family E member 1, alias PAI, plasminogen activator inhibitor, type I). Interestingly, low levels of the UPAR-encoding transcript were shown to characterize relapsed NB tumors [164].

**Figure 2:**
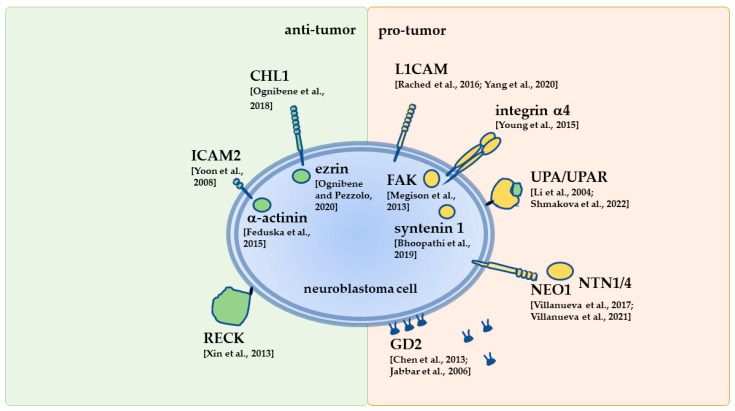
Examples of factors mediating interactions of NB cells with the ECM that affect malignancy (see Section 4, Section 5 and Section 6 for more detail) [81,86,87,98,99,102,103,105,106,120,122,148,149,160,163,164].

In Neuro 2a mouse NB cells, the involvement of UPAR in EGFR signal transduction to regulate proliferation or apoptosis, neuritogenesis was reported [165]. Additionally, the receptor was shown to interact with α3/α5β1 integrins to regulate adhesion [164]. Gutova et al. reported the enhanced migration of fetal-human-bone-marrow-derived mesenchymal stem cells and HB1.3F neural stem cells by conditioned media from cultures of NB1691 NB cells that ectopically over-expressed UPA or UPAR [166]. The downregulation of UPAR in Neuro2a cells increased cell size, reduced proliferation, impaired clonogenicity, and decreased adhesion, but stimulated the migration of cells. This was correlated with the upregulation of IL-6, N-cadherin, α-SMA, and vinculin (mesenchymal-phenotype related proteins), but a decrease in E-cadherin, occludin, and claudin-5 (epithelial markers), and decreased levels of nuclear phosphorylated-ERK1/2 and STAT1 [167]. Interestingly, in the absence of UPAR, UPA can be accumulated in the nucleus to regulate gene expression [167]. Moreover, in Neuro 2a cells, the downregulation of UPAR led to the upregulation of dormancy-related markers, i.e., phosphorylated P38 and P21, but downregulation of P53, as well as induced chemoresistance to cisplatin and doxorubicin. Finally, s.c. injection of cells with UPAR downregulation impaired primary tumor growth, but enhanced lung metastasis [164]. Figure 2 summarizes the discussed factors mediating interactions of NB cells with the ECM that affect malignancy.

## 7. Targeting Interactions of NB with ECM Components

Knowledge gathered about the molecular mechanisms of interactions between ECM components and NB cells stimulates the design of new approaches to target the tumor.

Integrins are key receptors which interact with the ECM. Recently, a tenascin-C-derived peptide (TNIIIA2) that activates integrin β1 was tested with retinoic acids, i.e., ATRA [168] or acyclic retinoid [169], and was shown to decrease the survival of IMR-32 cells, reduce protein levels of MYCN (by its proteasomal degradation) and aurora A, and impair tumor growth in mice. Interestingly, the integrin αvβ3 heterodimer was shown to bind thyroid hormones [170]. Thus, a dual specific agent was designed, containing a thyroid-hormone-derived antagonist of integrin αvβ3 and benzylguanidine to target NB via the norepinephrine transporter (NET). Both agents were linked via poly(ethylene glycol) (PEG) to aid solubility and tested to assess growth-suppressive and anti-angiogenic efficacy in NB models [171]. Cilengitide and SB273005, inhibitors of the αvβ3 and αvβ5 integrin heterodimers, were tested in NB cell cultures [144,172]. Cilengitide and etoposide showed synergistic cytotoxic effects on SK-N-BE(2) and NBL-S NB cells in vitro [172]. Additionally, SB273005 decreased the attachment of LA-N-5 and CHP-134 cells to vitronectin [144].

FAK is a pivotal component of the focal adhesion complex that transduces signals of activated integrins. Stafman et al. tested two FAK inhibitors, PF-573,228 and 1,2,4,5-benzentetraamine tetrahydrochloride (Y15), on PDX-derived NB cells in vitro [173]. The agents decreased the survival, proliferation, invasion, and migration, and reduced CSCLC populations in the models [173]. The finding that P53 and FAK are binding partners in NB cell lines laid a foundation for testing a combined treatment to inhibit FAK and activate P53 with PF-573,228 and nutlin-3, respectively, which resulted in synergistic cell death effects [174].

Peptide binding to NCAM (NTP) was used to deliver paclitaxel (PTX) with polyglutamic acid (PGA) as the carrier to NB cells [175]. The PGA–PTX–NTP conjugate was the most potent in inhibiting proliferation, migration, and in vivo tumor growth of IMR-32 cells, as compared with PGA–PTX and a conjugate in which NTP was replaced with a control peptide [175].

Antibodies and their conjugates can be utilized for the specific targeting of cancer cells. Application of the IgG1-iS18 mAb to target the laminin receptor on IMR-32 cells reduced their invasion into Matrigel and adhesion to laminin-1 [176]. Moreover, lorvotuzumab mertansine (IMGN901), an immunoconjugate consisting of the humanized mouse anti-NCAM huN-901 mAb conjugated to an anti-tubulin agent, was tested in a phase II clinical trial in relapsed or refractory childhood tumors including NB; however, limited clinical activity was reported [177].

Additionally, targeting NB via L1CAM with the application of a chimeric antigen receptor (CAR) that contains the scFv (i.e., single-chain fragment variable) of the CE7 mAb to modify T cells for adoptive immunotherapy is currently being developed [178].

Glypican 2 (GPC2) is over-expressed on NB cells [62], and it is a target for the tumor delivery of drugs in the form of immunoconjugates [179,180]. One such example could be the delivery of DNA-damaging pyrrolobenzadiazepine (PBD) dimers with applications of the D3-GPC2-IgG1 mAb that recognizes a tumor-specific epitope of GPC2 [179]. Another example is the application of a human VH single-domain antibody fragment (LH7), isolated using phage display technology, used for the preparation of an immunotoxin by conjugation to the *Pseudomonas* exotoxin (PE38) [180]. CARs targeting GPC2 were also constructed and shown to exhibit anti-NB efficacy in preclinical models [180,181]. Thus, the aforementioned LH7 Ab fragment was used to design a CAR T cell therapy [180]. An additional example is a CAR containing the scFv of the anti-GPC2 mAb (CT3) recognizing tumor-associated epitopes of GPC2 [181]. Moreover, CAR T cells targeting both GPC2 and B7-H3 (CD276) have been reported [182].

Last but not least, GD2 is being targeted with mAbs for the treatment of NB patients with dinutuximab [13], dinutixumab beta [14], and naxitamab [183]. The antibodies activate complement-dependent cytotoxicity (CDC) and antibody-dependent cell-mediated cytotoxicity (ADCC) to fight tumor cells. This stimulates the testing of further strategies in preclinical research and clinical trials to target GD2 with vaccines [184], immunotoxins, and CARs [185]. The developed approaches related to molecules involved in ECM–NB cell interactions are presented in Table 1.

## 8. Future Prospects and Conclusions

Neuroblastoma heterogeneity is manifested in the observed clinical diversity of the disease, and the elucidation of biological features of the tumor has laid the foundations for diagnosis, as well as risk and treatment stratification. Further research on interactions between ECM components and NB cells is needed to broaden our understanding of factors that play roles in the TME, and their involvement in signaling pathways regulating cell stemness and plasticity, especially in the context of metastasis, therapy resistance, dormancy, or relapse.

Interactions of NB cells with ECM components are complex. However, as reviewed in this article, several elements of the interplay have been identified and correlated with NB risk factors and prognosis. The prominent examples of such molecules are FAK, GPC2, L1CAM, NCAM, or GD2, which are now being exploited to target NB cells (see Section 7 and Table 1). It will be of great interest to further characterize anti-tumor effects of the aforementioned strategies, as well as their impact on non-cellular and cellular components of the TME, e.g., to establish if and how they modulate the immune landscape of NB. This view could be extended to the *MYCN* and *ALK* oncogenes, because they are frequently mutated in NB, have a well-established influence on TME, and are pivotal targets of clinically evaluated anti-tumor therapies. This could guide which approaches should be prioritized and further developed as drug candidates, and help to design treatment combinations to increase their anti-tumor efficacy, because the number of NB patients for participation in clinical trials is limited. Hopefully, this could yield new drugs or drug combinations to improve the survival of patients with high-risk neuroblastoma.

## Figures and Tables

**Table 1:** Examples of newly developed approaches to target neuroblastoma based on knowledge about interactions of the tumor cells with the TME (see Section 7 for more detail).

Therapy	Description	References
Tenascin C-derived peptide (TNIIIA2)	To activate integrin β1, tested in a combination with ATRA or acyclic retinoid	[168] [169]
BG-PEG_1600_-TAT	An antagonist of αvβ3 integrin linked via poly(ethylene glycol) to benzylguanidine	[171]
SB273005 Cilengitide	Inhibitors of αvβ3 and αvβ5 integrins combined with anti-GD2 14G2a mAb or etoposide	[144] [172]
PF-573,288	An inhibitor of FAK combined with nutlin 3 (to activate P53)	[174]
PGA–PTX–NTP	Targeting NCAM-positive cells with a peptide (NTP) to deliver paclitaxel (PTX)	[175]
IgG1-iS18	Anti-laminin receptor mAb	[176]
Lorvotuzumab mertansine (IMGN901)	Anti-NCAM mAb huN-901 conjugated to an anti-tubule agent	[177]
D3-GPC2-IgG1 combined with pyrrolobenzadiazepine	Anti-GPC2 mAb recognizing a tumor-specific epitope for the targeted delivery of a DNA-damaging agent	[179]
Immunotoxin of LH7 and PE38	The anti-GPC2 single domain Ab fragment LH7 linked to the *Pseudomonas* exotoxin (PE38)	[180]
CAR to modify T cells for adoptive immunotherapy	Contains the scFv of anti-L1CAM mAb CE7. Contains the anti-GPC2 Ab fragment LH7. Contains the scFv of anti-GPC2 mAb CT3.	[178] [180] [181]
GD2/GD3 vaccine	Keyhole limpet hemocyanin (KLH) conjugated to deliver active immunotherapy	[184]

## Data Availability

Data sharing not applicable.
